# Primary drug resistance at diagnosis of HIV-1 infection: a Portuguese cohort

**DOI:** 10.7448/IAS.17.4.19761

**Published:** 2014-11-02

**Authors:** Nuno Rocha Pereira, Raquel Duro, Carmela Piñero, Cristóvão Figueiredo, Ana Sofia Santos, Jorge Soares, Rosário Serrão, António Sarmento

**Affiliations:** Infectious Diseases, Centro Hospitalar São João, Porto, Portugal

## Abstract

**Introduction:**

Presence of viral mutations conferring resistance to antiretroviral drugs has potential impact on success of antiretroviral therapy (ART). The aim of this study was to describe the prevalence of resistance-associated mutations in HIV-infected patients without prior ART in a Portuguese cohort.

**Materials and Methods:**

Retrospective single-centre study of patients newly diagnosed with HIV-1 infection between 2006 and 2012. Resistance genotyping was obtained with HIV TRUGENE^®^ and Viroseq^®^ tests and the analysis of drug resistance was based on the Stanford University HIV Drug Resistance Database. Epidemiological data was also gathered. Continuous variables were summarized by mean and standard deviation, whereas categorical variables were presented as proportions. Comparison of proportions was performed with Chi square and Fisher exact test while means were compared with Student test. Statistical significance was assumed when p<0.05. Statistical analysis was performed with SPSS 21.0^®^.

**Results:**

Resistance testing was performed in 624 patients. General characteristics of the patients are summarized in Table 1. Mutations were found in 291 (46.6%) patients but resistance-associated mutations were present in 79 (12.7%) patients. Resistances to different drug classes were the following: NNRTIs-resistance in 42 (6.7%) patients; NRTIs-resistance in 19 (3.0%) patients; PIs-resistance in 30 (4.8%) patients. Only 10 (1.6%) patients presented simultaneous resistance-associated mutations to more than one class of drugs. There were no statistical significant differences between the years at which HIV-1 was diagnosed. Also no significant difference in the distribution of the parameters age, sex, CD4-cell count, and viral load, between groups with and without resistance was identified. Resistance-associated mutations were significantly more common in patients with non-B HIV-1 subtypes (15.4% vs 9.8%; p=0.048) and in those presenting with AIDS (18.2% vs 11.1%; p=0.03).

**Conclusions:**

Prevalence of resistance-associated mutations identified in this study was similar to those reported in similar studies from Western Europe. Knowledge about the epidemiology of primary resistance in our country is important in order to improve HIV care.

**Table 1:** General characteristics of the patients. Abbreviations used in the table: SD - Standard deviation; MSM - Men that have sex with men; CD4 - CD4+ T cell lymphocytes

Characteristics	n=624
Sex:	
Male	447 (71.6%)
Female	177 (28.4%)
Age (mean±SD)	40.92±13.38
Risk for HIV acquisition	
Heterosexual	436 (70.1%)
MSM	138 (22.2%)
Injecting drug user	46 (7.4%)
Others	2 (0.3%)
CD4-cell count /mL (mean±SD)	321±254
HIV-1 viral load copies/mL (mean±SD)	705725±1928167

